# Correction: Maternal exposure to sulfonamides and adverse pregnancy outcomes: A systematic review and meta-analysis

**DOI:** 10.1371/journal.pone.0263428

**Published:** 2022-01-27

**Authors:** Peixuan Li, Xiaoyun Qin, Fangbiao Tao, Kun Huang

The Funding statement is incorrect. The correct Funding statement is as follows: This work was supported by the National Natural Science Foundation of China (Grant 81872630 to KH); the Special Project Reproductive health, prevention, and control of major birth defects of National Key Research and Development Program (Grant 2016YFC1000204-2 to FT); and Sci-tech Basic Resources Research Program of China (Grant 2017FY101107 to KH). The funders had no role in study design, data collection and analysis, decision to publish, or preparation of the manuscript.
