# Supplementary figures and images for: Maternal exposure to sulfonamides and adverse pregnancy outcomes: A systematic review and meta-analysis
Source: PLoS One. 2020 Dec 2;15(12):e0242523. doi: 10.1371/journal.pone.0242523 (PMC7710089; doi:10.1371/journal.pone.0242523)

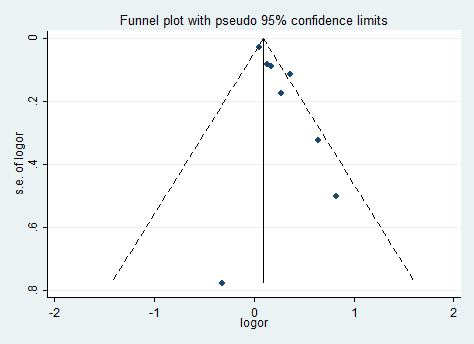

Supplement: S1 Fig — (TIF) [file pone.0242523.s003.tif]

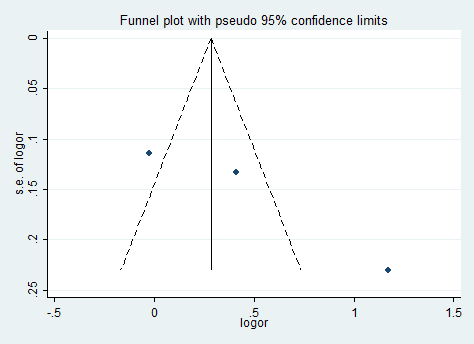

Supplement: S2 Fig — (TIF) [file pone.0242523.s004.tif]

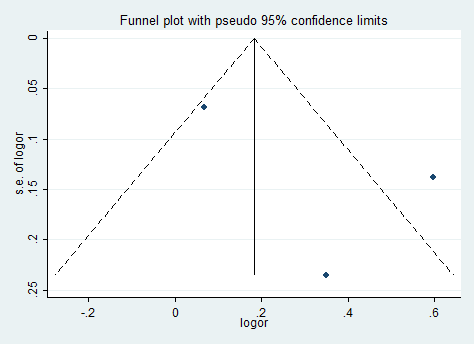

Supplement: S3 Fig — (TIF) [file pone.0242523.s005.tif]

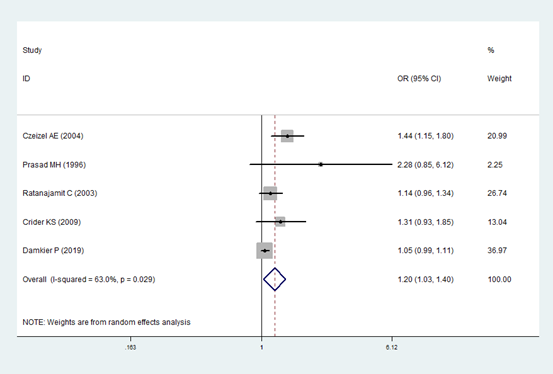

Supplement: S4 Fig — (TIF) [file pone.0242523.s006.tif]
